# The Efficacy and Safety of Miconazole Nitrate Mucoadhesive Tablets versus Itraconazole Capsules in the Treatment of Oral Candidiasis: An Open-Label, Randomized, Multicenter Trial

**DOI:** 10.1371/journal.pone.0167880

**Published:** 2016-12-15

**Authors:** Zhimin Yan, Xiaosong Liu, Yang Liu, Ying Han, Mei Lin, Wenmei Wang, Xiaobing Guan, Shengrong Zhu, Handong Zhang, Qintao Wang, Lihong Chou, Xinghao Zhu, Hong Hua

**Affiliations:** 1 Department of Oral Medicine, School and Hospital of Stomatology, Peking University, National Engineering Laboratory for Digital and Material Technology of Stomatology, Beijing, China; 2 Department of Oral Medicine, West China School of Stomatology, Sichuan University, Chengdu, China; 3 Department of Oral Medicine, Institute and Hospital of Dentistry, Nanjing University Medical School, Nanjing, China; 4 Department of Periodontics & Oral Medicine, Beijing Stomatological Hospital, Capital Medical University, Beijing, China; 5 Department of Stomatology, Tongji Hospital, Tongji Medical College, Huazhong University of Science and Technology, Wuhan, China; 6 Department of Stomatology, Union Hospital, Tongji Medical College, Huazhong University of Science and Technology, Wuhan, China; 7 Department of Periodontology, School of Stomatology, The Fourth Military Medical University, Xian, China; 8 Hospital of Stomatology, China Medical University, Shenyang, China; 9 Department of Stomatology, The First Affiliated Hospital of Wenzhou Medical University, Wenzhou, China; Cardiff University, UNITED KINGDOM

## Abstract

**Background:**

Oral candidiasis (OC) is a common oral fungal infection. Recently, miconazole mucoadhesive tablets have been gaining attention for OC treatment. Despite trials in patients with human immunodeficiency virus and cancer, evidence of its application in the large-scale, general population with OC is lacking. This study aimed to evaluate the efficacy and safety of miconazole nitrate mucoadhesive tablets in comparison with itraconazole capsules for OC treatment.

**Methods:**

The study was a randomized, open-label, parallel-armed, multicenter clinical trial. Totally, 343 patients diagnosed with OC, who met the inclusion criteria, were randomly assigned to either a treatment group that received miconazole nitrate mucoadhesive tablets (10 mg) once daily or a control group that received itraconazole capsules (100 mg QD) for 2 weeks, and were followed up for 2 weeks. The clinical cure, improvement of clinical symptoms/signs, mycologic cure, and safety were evaluated.

**Results:**

The mucoadhesive tablets (n = 171) did not show inferiority to itraconazole (n = 172) in the treatment of OC. At the end of the 14-day treatment, the clinical cure rates were 45.29% and 41.76% in the miconazole and itraconazole groups, respectively (P = 0.3472). At the end of the 14-day follow-up, the clinical cure rates were 51.18% and 41.76% in the miconazole and itraconazole groups, respectively (P = 0.0329). Adverse events occurred in 53 subjects (33 in the miconazole group and 20 in the itraconazole group). There was no statistical difference in the safety profile between miconazole and itraconazole (P = 0.0533). Thrombocytopenic purpura, although rare, occurred in one patient in the miconazole group and was considered a drug-related, severe adverse event.

**Conclusion:**

Miconazole nitrate mucoadhesive tablets may be as effective as systemic itraconazole capsule for OC treatment. Physicians should be cautious about thrombocytopenic purpura occurring as a rare and serious adverse event of miconazole nitrate.

**Trial Registration:**

Chinese Clinical Trial Register ChiCTR-TRC-13003935

## Introduction

Oral candidiasis (OC) is the most common human fungal infection of the oral cavity [1]. Although it is more common in patients with an impaired immune system, such as those undergoing chemotherapy for cancer and patients with human immunodeficiency virus (HIV) infection [2], elderly people who wear removable dentures or misuse antibiotics are prone to develop oral candida infection [3]. Currently, triazole antifungal drugs such as the commonly used fluconazole and itraconazole are frequently used to treat OC, but the prevalence of resistance and its side effects restrict its application [4, 5].

Miconazole is a synthetic imidazole antifungal agent that has been used to treat superficial fungal infections since the early 1970s. The only fungicidal azole—miconazole—has a broad-spectrum antifungal activity against the most frequent *Candida* species in oropharyngeal candidiasis, including *Candida albicans*, *C*. *tropicalis*, *C*. *glabrata*, *and C*. *krusei* [6, 7]. Moreover, its resistance in chronically treated patients is rarely reported. However, topical miconazole is used infrequently in patients with OC because of the requirement of multiple daily dosing [6]. To prolong the residence time of miconazole and maintain its fungicidal effect, a mucoadhesive buccal tablet with slow-release properties was developed [8]. Although previous studies have evaluated the effect of mucoadhesive tablets in the treatment of special subgroups of patients with HIV infection [9, 10] or head and neck cancer [11], the knowledge of its effectiveness and safety in the general management of patients with OC, which is a rather larger group, is still insufficient. Therefore, this study aimed to evaluate the effectiveness and safety of miconazole mucoadhesive tablets in the treatment of OC and establish a standard therapeutic program for its application.

## Materials and Methods

### Study design and trial registration

This study was a randomized, open-label, parallel-armed, positive-controlled clinical trial designed to compare the effectiveness of miconazole nitrate mucoadhesive tablet and itraconazole capsules in adult patients with OC. The study complies with the State Food and Drug Administration (SFDA) principles of China and Good Clinical Practice guidelines (GCP). The entire protocol was approved by the Human Research Ethics Committee of Peking University Health Center (2008–03) in October 2008. The trial has also been registered with the Chinese Clinical Trial Registry (ChiCTR-TRC-13003935). This retrospective registration was due to our unawareness of the international requirements of prospective registration for such kind of trials at that time. However, we ensure honestly that the delay of registration has little bearing on the quality and ethics of this study.

We confirm that all ongoing and related trials for this drug/intervention are registered. The study was guided by the CONSORT statement [12, 13].

### Study participants and settings

The inclusion criteria for all subjects were as follows: (1) age range, 18–70 years; (2) diagnosis of OC established on the basis of clinical manifestation and laboratory testing (smear test and/or fungi culture); and (3) negative reaction to pregnancy test in women and willingness to take effective birth control during the trial period. The exclusion criteria were as follows: (1) systemic fungal infections or chronic mucocutaneous candidiasis; (2) a history of a known allergy or intolerance to miconazole nitrate and/or itraconazole; (3) use of rifampicin, rifabutin, isoniazid, phenobarbital, phenytoin, methylprednisolone, carbamazepine, terfenadine, astemizole, or cisapride; (4) a history of psychological or other disorders making the patient unable to cooperate; (5) abnormal liver and kidney function; (6) a history of cardiac disorders such as ischemic heart failure; (7) a history of hematological diseases; (8) use of systemic or topical antifungal therapy within 2 weeks before study entry; (9) hyposalivation-related disease or drug-taking; (13) HIV infection; and (14) participation in other clinical trials within 4 weeks before study entry.

Patients who met the inclusion criteria in the nine research sites around China were enrolled in this trial from February 2009 to December 2012. Written informed consent was obtained from all subjects prior to participation.

### Sample size and randomization

Previous studies on the efficacy of itraconazole for treatment of OC [14,15,16] showed an efficacy rate of 85%. To achieve 80% power with a significance level of 10% (α-level, 5%; β-level, 20%; both one-sided) in this study, we required 158 participants per group with regard to the expected withdrawal rate of 20%. A stratified, block-randomization method was used for allocation of subjects to groups. In this randomized, open-label, positive-controlled clinical trial, the study drugs were packed and numbered according to the random-coding form and randomly allocated to each research site using concealed opaque envelopes. The study drugs were administered according to the assigned numbers, which were determined according to the visit sequence and study drug number sequence, and remained unchanged throughout the trial.

### Interventions

The treatment consisted of a 2-week treatment period and a 2-week follow-up period. Miconazole (10 mg) mucoadhesive tablets (Tibotec Pharmaceuticals Ltd., Co Cork, Ireland) were administered topically once daily, with the rounded side of the tablet applied to the canine fossa in the morning after brushing the teeth. A training program was conducted by the investigators for each patient prior to the drug administration, and instruction pamphlets were handed out to each patient for his/her reference. The patients were trained to apply the tablet by themselves at home once daily. As the tablet absorbs moisture from the mouth, it releases miconazole and slowly dissolves over time. The control group was administered itraconazole capsules (100 mg; Xian Janssen Pharmaceutical Ltd., Xian, China) once daily immediately after a full meal to improve its absorption. Follow-up visits were conducted at 7, 14, and 28 days.

### Outcomes measures

#### Primary outcome measures

The primary measures included evaluation of clinical symptoms (pain and burning sensation rating) and signs (pseudomembrane and erythema grade). The clinical symptoms (self-reported) were scored as 0 for no symptoms, 1 for mild symptoms, 2 for moderate symptoms, and 3 for severe symptoms. The signs of erythema or removable white plaque (extent of oral lesions) were scored as 0 (no lesions), 1 (lesions measuring < 0.5 cm^2^), 2 (lesions measuring 0.5–1 cm^2^), and 3 (lesions measuring > 1 cm^2^). Clinical cure was defined as the absence of signs and symptoms (score of 0) and confirmed by the investigator.

#### Secondary outcome measure

Mycological cure or improvement of OC was set as the secondary outcome (by smear test and fungi culture). Oral smears from the lesion were examined by microscopy using 10% potassium hydroxide. Subsequently, the fungi were cultured, and the colony-forming unit count was recorded and compared with the baseline data. Mycological eradication was defined as a negative smear test and negative culture (no growth of *Candida* spp.) at the test-of-cure visit.

#### Safety evaluation

Adverse reactions were defined as those that were reported as side effects by the subjects and/or reported as abnormal laboratory results post-treatment by the investigator. All laboratory reports that included data on blood pressure, electrocardiography, full blood count, and liver and kidney function tests were recorded and analyzed. Blood samples were obtained at the initial visit (prior to the medication intake, days -3 to 0) as the baseline data and at the 3rd visit after the final day of drug administration (day 14+1) to access its safety. If the patient reported abnormal blood results at the 3rd visit, additional blood tests were performed at the 4th visit (2-week follow-up visit after ceasing medication intake).

### Statistical analysis

We used SPSS 16.0 software for windows(SPSS Inc, Chicago, IL)for all data analysis. We followed the CONSORT guidelines for randomized controlled trials for analysis and reporting of the results [13].

A non-inferiority statistical approach was used. The margin of 10% was maintained with regard to both the statistic and clinical significance. All patients who received at least 1 dose of study medication were enrolled in the safety analyses. For early withdrawals, if the outcomes could not be measured, they were considered as non-responders. The trial data were processed and analyzed by an independent statistician, who was blinded to participant allocation.

Continuous data were compared using either the Student *t-*test or Mann-Whitney nonparametric test. For binary data, the two treatment groups were compared using the chi-square test or Fisher exact test. The Mantel-Haenszel test was used to evaluate ordinal quantitative data.

## Results

### Subject disposition

**Fig 1:**
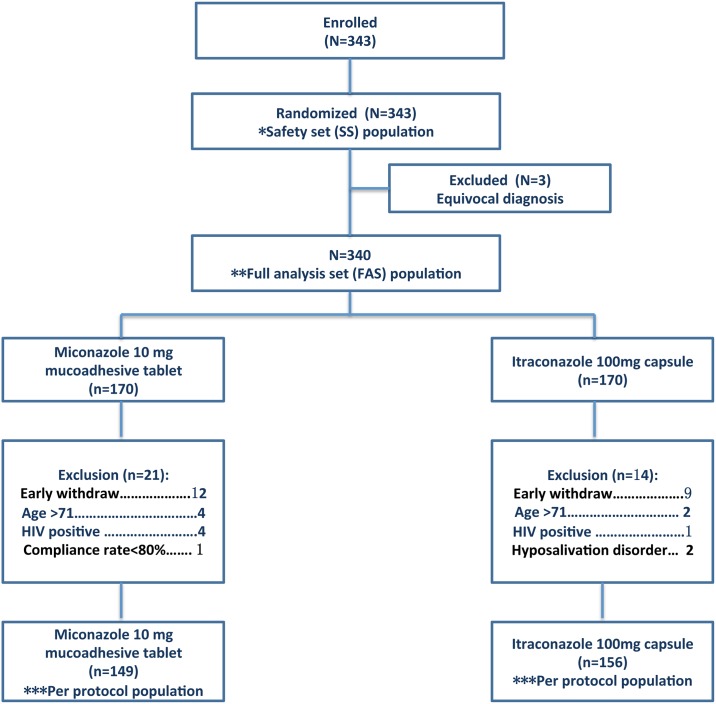
Patient randomization and disposition. *For safety analyses, the safety-set (SS) population was used. Safety population includes all randomized patients who take at least one dose of the study drug. **The full-analysis set (FAS) population is a trial population that is as close as possible to the general population for which a test treatment is intended. The FAS population can include individuals who fail to comply with the treatment protocol. *** The per-protocol population (PPS) is the subset of the FAS population that adhered to the treatment protocol, and consequently, excluding individuals who fail to comply with the treatment protocol.

Patients were recruited at nine hospital sites around China over a 46-month period. Based on the early stopping rules addressed in the protocol, the enrollment ended earlier due to a lower withdrawal rate than expected (20%). In total, 343 patients were enrolled in the study as the intent-to-treat population, and 3 of them were eventually excluded due to insufficient diagnosis, which resulted in a final population of 340 subjects as the full-analysis set/modified intent-to-treat population. The 340 subjects (170 in each group) included all subjects receiving trial medication at least once. The per-protocol population (excluding all major protocol violators; Fig 1) comprised 305 subjects (149 in the miconazole group and 156 in the itraconazole group). Patient disposition is summarized in Fig 1. The full-analysis set/modified intent-to-treat population was used to analyze the effectiveness of a trial treatment.

### Characteristics at baseline

There were no significant differences between the two groups with regard to age, sex, race, allergy history, underlying diseases, and duration of oral symptoms (Table 1). The underlying diseases in the medical background referred to the diseases patients might have, but were not listed in the exclusion criteria. The baseline characteristics of 340 subjects are presented in Table 2. The clinical symptoms (pain and burning) and signs (pseudomembrane and erythema) showed no significant difference between 2 groups.

**Table 1:** Demographics of subjects.

Baseline Characteristics* Subject Disposition	Miconazole Nitrate (N = 170)	Itraconazole (N = 170)	P value
Age (years, mean ± SD)	53.7±12.75	53.1±11.47	0.6583
Gender (M/F)	38/132	49/121	0.2148
Race (Han/others)	166/4	166/4	1.0000
Allergy history (Yes/No)	20/150	22/148	0.8693
Underlying diseases (Yes/No)	46/124	36/134	0.2538

*If not specified otherwise, the baseline results discussed are of the full-analysis set (FAS) population.

Abbreviations: SD, standard deviation

**Table 2:** Baseline characteristics of modified intent-to-treat patients who had oral candidiasis.

Symptoms/sign		Miconazole Nitrate (N = 170)	Itraconazole (N = 170)	P value
Duration of symptoms (mean ± SD) (day)		100.7±31.0	119.6±31.0	0.5836
**Pain**	No (0)	44 (25.9%)	41 (24.1%)	0.9981
	Mild (1)	88 (51.8%)	94 (53.5%)	
	Moderate (2)	33 (19.4%)	31 (18.8%)	
	Severe (3)	5 (2.9%)	4 (2.6%)	
**Burning**	No (0)	57 (33.5%)	54 (31.8%)	0.7800
	Mild (1)	75 (44.1%)	78 (45.9%)	
	Moderate (2)	36 (21.2%)	34 (20.0%)	
	Severe (3)	2 (1.2%)	4 (1.8%)	
**Pseudomemb-rane**	No (0)	89 (52.4%)	84 (49.4%)	0.6521
	Mild (1)	27 (15.9%)	30 (17.6%)	
	Moderate (2)	34 (20.0%)	35 (20.6%)	
	Severe (3)	20 (11.8%)	21 (12.4%)	
**Erythema**	No (0)	24 (14.1%)	26 (15.3%)	0.8341
	Mild (1)	46 (27.1%)	42 (24.7%)	
	Moderate (2)	57 (33.5%)	56 (32.9%)	
	Severe (3)	43 (25.3%)	46 (27.1%)	
**Total symptom/sign**		4.51±2.34	4.59±2.133	0.4676

Abbreviations: SD, standard deviation

### Efficacy analysis

At the end of the 28-day follow-up, the average decrease in the clinical symptom/sign score was 3.62 in the miconazole group and 3.28 in the itraconazole group (P = 0.3090). The 95% confidence interval (CI) for treatment difference was 7.10% (−0.12% to 20.12%). At the end of the 14-day treatment phase, the average decrease in the clinical symptom/sign score was 3.33 in the miconazole group and 3.16 in the itraconazole group (P = 0.7805). The 95% CI for treatment difference was 7.06% (−2.99% to 17.11%). At these two time points, miconazole nitrate was not statistically significantly inferior to itraconazole, considering the 10% maximal clinically allowable difference (P = 0.005). The clinical efficacy, including the clinical cure (primary variable) and mycological eradication (secondary variable) at the end of treatment and at the end of follow-up, is presented in Table 3.

**Table 3:** Comparison of the therapeutic efficacy of miconazole and itraconazole between groups.

Efficacy		Miconazole Nitrate (N = 170)	Itraconazole (N = 170)	Treatment difference (95%CI)^a^	P value
**Primary variable 1 At end of treatment**	Clinical cures, n (%)	77 (45.29%)	71(41.76%)	3.53%	0.3472
	95% CI of response rate	(37.66%; 53.10%)	(34.26%; 49.56%)	(-7.00%; 14.06%)	
**Primary variable 2 At end of follow-up**	Clinical cures, n(%)	87(51.18%)	71(41.76%)	9.41%	0.0329
	95% CI of response rate	(43.41%; 58.91%)	(34.26%; 49.56%)	(-1.14%; 19.97%)	
**Secondary variable 1 At end of treatment**	Mycological eradication, n (%)	55(39.86%)	32(22.54%)	17.32%	0.0001
	95% CI of response rate	(31.62%; 48.53%)	(15.95%; 30.30%)	(6.65%; 27.99%)	
**Secondary variable 2 At end of follow-up**	Mycological eradication, n (%)	40(28.99%)	32(22.54%)	6.45%	0.1014
	95% CI of response rate	(21.58%; 37.31%)	(15.95%; 30.30%)	(-3.77%; 16.67%)	

^a^ If not specified otherwise, the efficacy results discussed are of the full-analysis set (FAS) population.

Abbreviations: SD, standard deviation; CI, confidence interval

If clinical cure was achieved in combination with mycological eradication, we defined the condition as “cured.” A total of 53 patients (31.36%) in the miconazole group and 49 patients (29.17%) in the itraconazole group were cured at the end of treatment. Interestingly, 14 days later, there was an increasing trend of 71 patients (42.01%) and 54 patients (32.14%) cured in the miconazole and itraconazole groups, respectively, with no significant difference in the effectiveness between groups at the 14- and 28-day time points (P = 0.0712).

### Safety analysis

In total, adverse events occurred in 53 subjects (33 in the miconazole group and 20 in the itraconazole group, Table 4). There was no statistical difference in the safety profile between the miconazole and itraconazole groups (P = 0.0533). Upon evaluation, 25 of the 53 cases of adverse effects were defined as drug-related adverse effect. Of the 25 cases, 15 belonged to the miconazole group and 10, to the itraconazole group. The incidence of drug-related adverse events (8.8% in the miconazole group vs 5.8% in the itraconazole group) was similar between the two treatment groups.

**Table 4:** Summary of overall safety data.

AE	Miconazole Nitrate (N = 171)	Itraconazole (N = 172)	Total (N = 343)	P value
Subjects with ≥1 AE	33 (19.3%)	20 (11.6%)	53 (15.5%)	0.0533
Subjects with ≥1 ADE	15 (8.8%)	10 (5.8%)	25 (7.3%)	0.3078
Subjects with ≥1 drug-related SAE	2 (1.2%)	0 (0%)	2 (0.6%)	0.2478
Subjects with ≥1 AE ceasing drug use	1 (0.6%)	1(0.6%)	2 (0.6%)	1.0000

Abbreviations: AE, adverse events; ADE, adverse drug events; SAE, serious adverse events

Two serious adverse events were reported and both occurred in the miconazole group: one of them (thrombocytopenic purpura) was defined as drug-related, and the patient recovered with good prognosis. Two subjects ceased drug use due to adverse effects: one of the subjects who had acute gastroenteritis belonged to the itraconazole group and the other one who had thrombocytopenic purpura belonged to the miconazole group. Additional adverse events included dizziness (5 patients), abnormal urinary albumin level (3 patients), and abnormal aspartate transaminase level (3 patients) in the miconazole group; furthermore, white blood cell reduction (4 patients) and an increase in the alanine aminotransferase level were more frequently observed in the itraconazole group. All adverse events that occurred are listed in Table 5.

**Table 5:** Summary of adverse events.

Adverse event	Miconazole Nitrate (N = 171)				Itraconazole (N = 172)			
	*%*	*Severity*			*\%*	*Severity*		
		*Mild*	*Moderate*	*Severe*		*Mild*	*Moderate*	*Severe*
**Abnormal lab testing**	6(3.5%)	5(83.3%)	1(16.7%)	-	6(3.5%)	5(83.3%)	1(16.7%)	-
WBC ↓	2(1.2%)	2(100.0%)	-	-	4(2.3%)	4(100.0%)	-	-
AST ↑	1(0.6%)	-	1(100.0%)	-	2(1.2%)	2(100.0%)	-	-
Serum bilirubin ↑	2(1.2%)	2(100.0%)	-	-	1(0.6%)	1(100.0%)	-	-
ALT elevation ↑	-	-	-	-	2(1.2%)	1(50.0%)	1(50.0%)	-
Urinary albumin (+)	1(0.6%)	1(100.0%)	-	-	1(0.6%)	1(100.0%)	-	-
γ-GT ↑	-	-	-	-	1(0.6%)	-	1(100.0%)	-
Lymphocyte% ↑	-	-	-	-	1(0.6%)	1(100.0%)	-	-
Urinary RBC (+)	-	-	-	-	1(0.6%)	1(100.0%)	-	-
Neutrophil%↓	-	-	-	-	1(0.6%)	1(100.0%)	-	-
PLT ↓	-	-	-	-	1(0.6%)	1(100.0%)	-	-
**Gastrointestinal symptoms**	5(2.9%)	5(100.0%)	-	-	3(1.7%)	2(66.7%)	1(33.3%)	-
Xerostomia	1(0.6%)	1(100.0%)	-	-	2(1.2%)	2(100.0%)	-	-
Upper abdomen pain	2(1.2%)	2(100.0%)	-	-	1(0.6%)	-	1(100.0%)	-
Nausea	1(0.6%)	1(100.0%)	-	-	-	-	-	-
**Abdominal discomfort**	1(0.6%)	1(100.0%)	-	-	-	-	-	-
Localized pruritus	1(0.6%)	1(100.0%)	-	-	-	-	-	-
Oropharyngeal discomfort	-	-	-	-	1(0.6%)	1(100.0%)	-	-
Vomiting	-	-	-	-	1(0.6%)	-	1(100.0%)	-
**Neuropathic symptoms**	4(2.3%)	4(100.0%)	-	-	1(0.6%)	1(100.0%)	-	-
Dizziness	3(1.8%)	3(100.0%)	-	-	-	-	-	-
Hypoesthesia	1(0.6%)	1(100.0%)	-	-	1(0.6%)	1(100.0%)	-	-
**Musculoskeletal symptoms**	-	-	-	-	2(1.2%)	1(50.0%)	1(50.0%)	-
Back pain	-	-	-	-	1(0.6%)	-	1(100.0%)	-
Joint pain	-	-	-	-	1(0.6%)	1(100.0%)	-	-
**Ophthalmologic symptoms**	1(0.6%)	1(100.0%)	-	-	1(0.6%)	1(100.0%)	-	-
Dry eyes	-	-	-	-	1(0.6%)	1(100.0%)	-	-
Paresthesia of eyes	1(0.6%)	1(100.0%)	-	-	-	-	-	-
**Respiratory symptoms**	1(0.6%)	1(100.0%)	-	-	-	-	-	-
Orofacial pain	1(0.6%)	1(100.0%)	-	-	-	-	-	-
**Skin symptoms**	-	-	-	-	1(0.6%)	1(100.0%)	-	-
Rash	-	-	-	-	1(0.6%)	1(100.0%)	-	-
**Hematological system disorder**	1(0.6%)	-	1(100.0%)	-	-	-	-	-
Thrombocytopenic purpura	1(0.6%)	-	1(100.0%)	-	-	-	-	-

## Discussion

The Infectious Diseases Society of America [17] and the Centers for Disease Control and Prevention [18] endorse the use of topical antifungal agents as the first-line drug for the treatment of mild cases of OC. However, multiple dosage daily and oral discomfort of topical agents limit its application in the clinical management of OC. The use of miconazole nitrate-sustained release mucoadhesive tablet as a topical antifungal therapy has been proposed as a promising and safe option to treat OC. Since evidence regarding its application in large-scaled general OC populations is lacking, a clinical trial is required to confirm the efficacy and safety of miconazole nitrate-sustained release mucoadhesive tablet treatment in comparison with oral itraconazole capsule for systemic treatment.

The results of the current multicenter, comparative, open-label, randomized study demonstrated that mucosal administration of miconazole (10 mg) mucoadhesive tablet was an efficacious and safe alternative to systemic oral antifungal agents for the treatment of OC. The subjects were randomized to receive either topical therapy using a 10-mg miconazole nitrate mucoadhesive buccal tablet once daily or systemic therapy using 100 mg itraconazole once daily for 14 consecutive days. Antifungal treatment regimen typically ranges from 7 to 14 days. Van Roey J et al. reported clinical cure after a 7-day treatment regimen of 10 mg miconazole nitrate mucoadhesive buccal tablet in HIV-positive patients [9]. However, in our study, no clinical cure was observed at 7 days after treatment.

The clinical response rate at the end of treatment and at the end of the 14-day follow-up demonstrated that the miconazole nitrate mucoadhesive tablet was not significantly inferior to the itraconazole capsule. After stopping the medication for 14 days, the miconazole nitrate group showed a tendency toward a higher “cure” rate than the itraconazole group (42.01% vs. 32.14%, respectively), although the difference was not significant at the end of the treatment (31.36% vs. 29.17%, respectively). This might be related to the slow-release system with high, sustained salivary concentrations obtained with the mucoadhesive tablets [19]. In this study, we defined “cure” as clinical cure combined with mycological eradication. However, some studies have shown no definite relation between clinical success or clinical cure and mycologic cure, and this could be because *Candida* is a commensal host of the oral mucosa [20]. Nevertheless, in this study, we were also interested in the fungicidal effect of miconazole nitrate, although mycologic eradication is not an objective of typical antifungal treatment.

In our current study, there was a serious or unexpected adverse effect related to the use of the miconazole nitrate mucoadhesive tablet. One subject developed thrombocytopenic purpura, which was considered a drug-related serious adverse effect. The platelets reduced to zero in this case. Between January 2004 and October 2012, 4 individuals using Daktarin ointment (miconazole nitrate) ointment reported thrombocytopenic purpura to the FDA. Although rare, physicians still need to be cautious about this adverse effect and take precautions to closely monitor the affected patients.

In this study, we demonstrated that the 10 mg topical miconazole nitrate mucoadhesive tablet is not inferior to the systemic itraconazole capsule in the treatment of general OC. It allows a once-daily topical treatment option and provides a similar efficacy rate as oral agents; it may also contribute to good patient compliance. Its favorable efficacy noted in this study suggests that the miconazole nitrate mucoadhesive tablet might be an important routine treatment regimen for treating OC in clinical practice. Moreover, miconazole nitrate mucoadhesive tablets have some advantages such as the ability to be used in patients with dry mouth or severe hyposalivation (where tablets are difficult to dissolve) and in patients wearing upper denture including the canine region.

Despite our important findings, this study has a few limitations that should be considered. This was a prospective, randomized, controlled but open-label study. The open-label protocol could have introduced bias in the study. In addition, the follow-up period was short, owing to which, we were not able to ascertain the maintenance of efficacy. Moreover, our results would be more reflective it the treatment outcomes were stratified and analyzed by the severity of OC.

## Conclusions

This study illustrated the efficacy and safety of the miconazole nitrate (10 mg) mucoadhensive tablet in the management of general OC patients via a well-designed multicenter clinical trial. The miconazole nitrate (10 mg) mucoadhesive tablet daily was as effective as the oral itraconazole (100 mg) regimen in treating patients with OC in nine centers around China. Furthermore, physicians should be cautious about the adverse effect of thrombocytopenic purpura, which may occur as a rare but serious side effect. This study provides strong evidence for the efficacy of miconazole nitrate and may be applicable in clinical practice.

## Supporting Information

S1 File Additional recorded information of the clinical trial. (PDF)

S2 File Scheme of the trial protocol. (DOCX)

S3 File CONSORT Checklist. (PDF)
