# Supplementary material for: The Efficacy and Safety of Miconazole Nitrate Mucoadhesive Tablets versus Itraconazole Capsules in the Treatment of Oral Candidiasis: An Open-Label, Randomized, Multicenter Trial
Source: PLoS One. 2016 Dec 15;11(12):e0167880. doi: 10.1371/journal.pone.0167880 (PMC5157999; doi:10.1371/journal.pone.0167880)
Supplement: S1 File — (PDF) [file pone.0167880.s001.pdf]

An evaluation on the efficacy and safety of topical oral miconazole nitrate paste in the treatment of oral candidiasis: a randomized, open label, positive controlled, multicenter clinical trial

Registration number:	ChiCTR-TRC-13003935
Date of releasing the registration number:	2013/12/03
Registration Status:	Retrospective registration
Public title:	An evaluation on the efficacy and safety of topical oral miconazole nitrate paste in the treatment of oral candidiasis: a randomized, open label, positive controlled, multicenter clinical trial
Scientific title:	An evaluation on the efficacy and safety of topical oral miconazole nitrate paste in the treatment of oral candidiasis: a randomized, open label, positive controlled, multicenter clinical trial
Secondary ID:	

Applicant:	Zhimin Yan	Study leader:	Hong Hua
Applicant telephone:	+86 10 82195349	Study leader's telephone:	+86 10 82195349
Applicant Fax:	+86 10 62173402	Study leader's fax:	+86 10 62173402
Applicant E-mail:	yzhimin@gmail.com	Study leader's E-mail:	honghua1968@yahoo.com.cn
Applicant website(voluntary supply):		Study leader's website(voluntary supply):	
Applicant address:	22 Zhongguancun Nandajie, Haidian District, Beijing	Study leader's address:	22 Zhongguancun Nandajie, Haidian District, Beijing
Applicant postcode:	100081	Study leader's postcode:	100081
Applicant's institution:	Peking University School and Hospital of Stomatology		

Approved by ethic committee:	Yes		
Approved No. of ethic committee:	（2009）伦申第（03）号	Approved file of Ethical Committee:	查看附件View
Name of the ethic committee:	Ethic Committee of Peking University Health Science Center		
Date of approved by ethic committee:	2009/09/15		

Primary sponsor:	Peking University School and Hospital of Stomatology
Primary sponsor's address:	22 Zhongguancun Nandajie, Haidian District, Beijing

Secondary sponsor:

Country:	China	Province:	Guangdong	City:	Shenzhen
Institution hospital:	JIAN AN PHARMACEUTICAL LIMITED	Address:	No.1016 Shangbu Middle Road, Futian District		

Source(s) of funding:	Hangzhou Tigermed Consulting Co., Ltd
Target disease:	Oral Candidiasis
Target disease code:	
Study type:	Interventional
Study phase:	Phase III
Objectives of Study:	To evaluate the efficacy and safety of topical oral miconazole nitrate paste in the treatment of oral candidiasis.
Study design:	Randomized parallel control
	The inclusion criteria were: 1) age range of 18-70 years; 2) diagnosis of oral candidiasis was established based on clinical manifestation and

Inclusion criteria	laboratory testing (smear test and fungi culture); 3) patients with positive smear test are eligible for instant enrollment; 4) positive rate of fungi culture>80%; 5) negative reaction to pregnancy test in women and willing to take effective method of birth control in the period of trial; 6) voluntarily participating and Informed Consent Form being signed.
Exclusion criteria:	1) diagnosis of cryptococcosis or other systemic fungal infections; 2) a history of a known allergy or intolerance to miconazole nitrate and/or itroconazole; 3) use of rifampicin, rifabutin, isoniazid, phenobarbital, phenytoin, methylprednisolone, carbamazepine, terfenadine, astemizole or cisapride; 4) history of pregnancy or breast-feeding; 5) history of psychological disorder that enable to cooperate; 6) hepaticinsufficiency with serum aminotransferase and total bilirubin levels at 1.5 times the upper limit of normal, or active clinical signs 2 months prior to the trial; 7) serum creatinine levels at 1.5 times the upper limit of normal; 8) history of cardiac insufficiency such as ischemic heart failure; 9) history of hematological diseases; 10) use of systemic or topical antifungal therapy within 2 weeks before study entry (patients received topical treatment with miconazole nitrate or nystatin cream or suppositor remain eligible for enrollment in the study); 11) diagnosis of chronic mucocutaneous candidiasis; 12) hyposalivation related disease or drug-taking; 13) HIV positive; 14) history of participating other clinical trials within 4 weeks before study entry.
Study execute time:	From2009-2-26To 2012-12-5

Interventions:

Group:	miconazole nitrate group	Sample size:	190
Intervention:	miconazole nitrate sustained release oral paste for topical use once a day	Intervention code:	
Group:	Itroconazole group	Sample size:	190
Intervention:	Itroconazole capsule 100mg QD P.O.	Intervention code:	

Countries of recruitment and research settings:

Country:	China	Province:	Beijing	City:	
Institution hospital:	Peking University School and Hospital of Stomatology	Level of the institution:	Tertiary A hospital		
Country:	China	Province:	Sichuan	City:	Chengdu
Institution hospital:	West China School of Stomatology, Sichuan University	Level of the institution:	Tertiary A hospital		
Country:	China	Province:	Jiangsu	City:	Nanjing
Institution hospital:	Institute and Hospital of Dentistry, Nanjing University Medical School	Level of the institution:	Tertiary A hospital		
Country:	China	Province:	Hubei	City:	Huhan
Institution hospital:	Tongji Hospital, Tongji Medical College, Huazhong University of Science and Technology	Level of the institution:	Tertiary A hospital		
Country:	China	Province:	Hubei	City:	Wuhan
Institution hospital:	Wuhan Union Hospital, Huazhong University of Science and Technology	Level of the institution:	Tertiary A hospital		
Country:	China	Province:	Shanxi	City:	Xi'an
Institution hospital:	College of Stomatology, the Fourth Military Medical University	Level of the institution:	Grade III		
Country:	China	Province:	Liaoning	City:	Shenyang
Institution hospital:	Hospital of Stomatology, China Medical University	Level of the institution:	Tertiary A hospital		
Country:	China	Province:	Beijng	City:	Beijing
Institution hospital:	Stomatological Hospital, Capital Medical University	Level of the institution:	Tertiary A hospital		

Outcomes:

Outcome:	Clinical symptoms rating (Pain and burning sensation level)
Outcome:	Clinical sign rating(pseudomenbran and erythema)
Outcome:	candida elimination rate
Outcome:	ECG
Outcome:	CBC
Outcome:	Biochemical Examination

Collecting sample(s) from participants:	Sample Name:	Blood	Tissue:	
	Fate of sample:	Destruction after use	Note:	
	Sample Name:	Saliva	Tissue:	
	Fate of sample:	Destruction after use	Note:	

Recruiting status:	Completed	Participant age:	Min age 19 years<br>Max age 70 years
Randomization Procedure (please state who generates the random number sequence and by what method):	computer	Gender:	Both
Blinding:			
Calculated Results ater the Study Completed:			
Organizer institution (leader institution):			
Data collection Institution:			
Data management Institution:			
Data analysis Institution:			

Return to list

Home | About ChiCTR | Trial Search | Document | Reg guide | Question

The world health organization international clinical trials registered organization registered platform
Copyright(c) (2005 - 2011) Chictr.org.All rights reserved. The Chinese clinical test registration center
Support: 543 social work center
蜀ICP备07505307号
Tips: it is recommended to use more than IE8.0 widescreen display resolution version using system.
8032724
11338
473
站长统计
