# Supplementary material for: The Efficacy and Safety of Miconazole Nitrate Mucoadhesive Tablets versus Itraconazole Capsules in the Treatment of Oral Candidiasis: An Open-Label, Randomized, Multicenter Trial
Source: PLoS One. 2016 Dec 15;11(12):e0167880. doi: 10.1371/journal.pone.0167880 (PMC5157999; doi:10.1371/journal.pone.0167880)
Supplement: S2 File — (DOCX) [file pone.0167880.s002.docx]

**The Efficacy and Safety of Miconazole Nitrate 10mg Mucoadhesive Tablets Verus Itraconazole Capsules in the Treatment of Oral Candidiasis: A randomized, open label, multicenter phase III trial**

RESEARCH SCHEME

Scheme No.: TG0817TIB

Version 3.0

Chinese Registration No.: ChiCTR-TRC-13003935

Research Responsible Department: Peking University School and Hospital of Stomatology

Research leader: Professor Hong Hua

 Date: January 2009

**Abstract of Research Scheme**

| Research topic | The Efficacy and Safety of Miconazole Nitrate 10mg Mucoadhesive Tablets Verus Itraconazole Capsules in the Treatment of Oral Candidiasis: A randomized, open label, multicenter phase III trial |
| --- | --- |
| Research objectives | To evaluate the Efficacy and Safety of Miconazole Nitrate 10mg Mucoadhesive Tablets in comparison with Itraconazole Capsules |
| Research design | A randomized, open label, multicenter phase III trial |
| Inclusion criteria | The inclusion criteria: 1) age range of 18-70 years; 2) diagnosis of oral candidiasis was established based on clinical manifestation and laboratory testing (smear test and/or fungi culture); 3) negative reaction to pregnancy test in women and willing to take effective method of birth control in the period of trial. |
| Exclusion criteria | The exclusion criteria: 1) systemic fungal infections or chronic mucocutaneous candidiasis; 2) a history of a known allergy or intolerance to miconazole nitrate and/or itraconazole; 3) use of rifampicin, rifabutin, isoniazid, phenobarbital, phenytoin, methylprednisolone, carbamazepine, terfenadine, astemizole or cisapride; 4) history of psychological or other disorder that unable to cooperate; 5) Abnormal liver and kidney function; 6) history of cardiac disorders such as ischemic heart failure; 7) history of hematological diseases; 8) use of systemic or topical antifungal therapy within 2 weeks before study entry; 9) hyposalivation related disease or drug-taking; 13) HIV infection; 14) history of participating other clinical trials within 4 weeks before study entry. |
| Index of efficacy | Primary: Evaluation of clinical symptoms (pain and burning sensation rating), signs (pseudomembrane and erythema grade).  Scondary: Mycological eradication of organism (by smear test and fungi culture) |
| Index of Safety | Bood pressure, electrocardiography, full blood count and liver and kidney function |
| Dosage regimen | Miconazole Nitrate 10mg Mucoadhesive Tablets Itraconazole 100mg Capsules |
| Course of treatment | 2 weeks treatment and 2 weeks follow-up |

**Research institutions and investigators**

| **Institutions** | **Investigators** |
| --- | --- |
| Peking University School and Hospital of Stomatology | Hong Hua  Zhimin Yan  Xiaosong Liu  Yang Liu  Ying Han |
| West China School of Stomatology, Sichuan University, | Mei Lin |
| Institute and Hospital of Dentistry, Nanjing University Medical School | Wenmei Wang |
| Stomatological Hospital, Capital Medical University, | Xiaobing Guan |
| Tongji Hospital, Tongji Medical College, Huazhong University of Science and Technology | Shengrong Zhu |
| Union Hospital, Tongji Medical College, Huazhong University of Science and Technology | Handong Zhang |
| School of Stomatology, The Fourth Military Medical University | Lihong Chou |
| The First Affiliated Hospital of Wenzhou Medical University | Xinghao Zhu |

**TABLE OF CONTENT(S)**

1.BACKGROUND............................................................................... 1 2.OBJECTIVES.................................................................................... 2 3.RESEARCH METHODS ................................................................. 2

3.1 RESEARCH DESIGN ...................................................................... 3

3.2 SAMPLE SIZE ................................................................................3 3.3 RANDOMIZED DESIGN ................................................................. 3 3.4 BLINDING LEVEL......................................................................... 3

4. RESEARCH SUBJECTS................................................................. 3

4.1 DIAGNOSTIC CRITERIA ................................................................3 4.2 INCLUSION CRITERIA.................................................................. 4

4.3 EXCLUSION CRITERIA ................................................................ 4

4.4 RECRUITMENT............................................................................. 5

5. INDEX OF EFFICACY ................................................................. 5

5.1 PRIMARY OUTCOME .....................................................................5

5.2 SECONDARY OUTCOM................................................................. 6

5.3 SAFETY MONITORING.................................................................. 6

6. TRAIL DISCONTINUATION OR MODIFICATION................ 7

6.1 WITHDRAW DECIDED BY RESEARCHER ..................................... 7

6.2 SUBJECT WITHDRAW RESEARCH VOLUNTARILY........................ 7

6.3 SITUATION TO DISCONTINUE RESEARCH ....................................8

7. INVESTIGATIONAL DRUGS .................................................... 8

7.1 NAME, SOURCE OF INVESTIGATIONAL DRUG ............................ 8

7.2 DRUG INVENTORY AND RECOVERY ........................................... 9 7.3 DRUG PRESERVATION ................................................................9 7.4 DRUG DISPENSATION AND RECOVERY....................................... 9

7.5 DRUG INVENTORY AND MANAGEMENT ..................................... 9

8. THERAPEUTIC REGIMEN AND RESEARCH PROCESS....... 10

8.1 DOSAGE REGIMEN..................................................................... 10

8.2 ADMINISTRATION INSTRUCTION................................................10

8.3 RESEARCH PROCESS.................................................................. 11

9. OBSERVATION OF ADVERSE EVENT ................................... 13

9.1 DEFINITION ............................................................................... 13

9.2 CRITERIA FOR JUDGING INTENSITY OF ADVERSE EVENT ........... 14

9.3 JUDGING STANDARD OF THE RELATION BETWEEN ADVERSE EVENTS AND INVESTIGATIONAL DRUG............................................ 14

9.4 ABNORMAL LABORATORY RESULTS......................................... 15

9.5 FOLLOW-UP AND ADVERSE EVENT RECORD ............................... 15

10. DATA MANAGEMENT .....................................................................15

11. STATISTICAL ANALYSIS......................................................... 16

11.1 ANALYSIS SET.......................................................................... 16

11.2 STATISTICAL ANALYSIS METHOD............................................. 17

12. ETHICS……………………….. .................................................. 17

12.1 ETHICS REVIEW........................................................................ 17 12.2 INFORMED CONSENT FOR SUBJECTS............................................ 17

12.3 ETHICS AND TRIAL REGISTRATION...............................................18

13. REFERENCES ............................................................................. 18

**The Efficacy and Safety of Miconazole Nitrate 10mg Mucoadhesive Tablets Verus Itraconazole Capsules in the Treatment of Oral Candidiasis: A randomized, open label, multicenter phase III trial**

**1. Background**

Oral candidiasis is a common form of oral fungal disease. Terrible oral hygiene, xerostomia, HIV infection, and radiation therapy for head and neck cancer have resulted in a significant proportion of the population suffering from oral candidiasis. The accepted treatment for oral candidiasis is the use of polyenes, such as and not limited to amphotericin B and nystatin, and azoles including fluconazole, miconazole, itraconazole, and clotrimazole. The CDC, IDSA, NIH, and HIVMA emphasize that topical antifungal therapy should serve as first-line therapy for mild OPC. Currently available topical agents involving nystatin, amphotericin B and clotrimazole have limitations, such as short retention time on the oral mucosa and the need for multiple applications each day. Moreover, many topical agents contain sugar, which may affect the oral cavity and patients with systemic disorders. Fungal resistance is also a significant concern and common problem .

Miconazole is a synthetic imidazole antifungal regimen that has been used for nearly 40 years to effectively and safely treat superficial fungal infections. Miconazole can damage the integrity of the fungal cell membrane, alter fungal adherence, and inhibit the formation of germ tubes and mycelia. Miconazole has potent broad-spectrum activity against many *Candida* species, including *Candida albicans* (*C. albicans*)*, Candida dubliniensis* (*C. dubliniensis*)*, Candida parapsilosis* (*C. parapsilosis*)*, Candida glabrata* (*C. glabrata*)*, Candida famata* (*C. famata*) and *Candida tropicalis* (*C. tropicalis*). Furthermore, miconazole is also effective against species of *Candida* that are resistant to fluconazole (*C. albicans*, some *C. glabrata*). The recent availability of miconazole offers an alternative based on its various topical formulations, broad-spectrum activity, and less resistance. Various up-to-date topical formulations of miconazole have been used to treat oral candidiasis, including miconazole buccal tablets, miconazole chewing gum, miconazole oral gel, and miconazole lacquer.

However, although there were studies evaluating the effect of mucoadhesive tablet in treating special subgroups of patients with HIV infection or with head and neck cancer, the knowledge of its effectiveness and safety in the management of general oral candidiais patients, which is a rather larger group, is still insufficient. Therefore the aim of this study is to evaluate the effectiveness and safety of miconazole mucoadhesive tablet in treating oral candidiasis and to establish a standard therapeutic program for its application.

**2. Objectives**

To evaluate the the efficacy and safety of miconazole nitrate 10mg mucoadhesive tablets in comparison with itraconazole 100mg Capsules in the treatment of oral candidiasis.

**3. Research Methods**

3.1 Research design

This study is a randomized, parallel-armed, positive-controlled, open label clinical trial comparing mucoadhesive miconazole tablet and itroconazole capsules in adult patients with oral candidiasis. The study complies with the State Food and Drug Administration (SFDA) principles of China and Good Clinical Practice guidelines (GCP). Its reporting will be guided by the CONSORT statement.

3.2 Sample size

In the present data of studies on the efficacy of itroconazole treating oral candidiasis, an 85% efficacy rate was assumed. To achieve 80% power with a significance level of 10%(α level, 5%; βlevel, 20%; both 1-sided), 158 participants per group would be required. To compensate for a potential 20% dropout rate, a sample size of 380 participants in total will be applied for this study.

3.3 Randomized design

Randomization will be carried out using block randomized sequences generated by computer. In this study, sealed envelope system will be used for randomization. In this participating investigators are given randomly generated treatment allocations within sealed opaque envelopes. Once a participant has consented to enter a trial an envelope is opened and the participant is then assigned an ID code and offered the allocated treatment regimen.

3.4 Blinding level

In this open label study, participants, investigators and outcome assessors will not remain blinded to the treatment.

**4. Research subjects**

4.1 Diagnostic criteria

Diagnosis of oral candidiasis was established based on clinical manifestation and laboratory testing (smear test and/or fungi culture)

4.2 Inclusion criteria

1) age range of 18-70 years; 2) diagnosis of oral candidiasis was established based on clinical manifestation and laboratory testing (smear test and/or fungi culture); 3) patients with positive smear test are eligible for instant enrollment; 4) positive rate of fungi culture>80%; 5) negative reaction to pregnancy test in women and willing to take effective method of birth control in the period of trial; 6) voluntarily participating and Informed Consent Form being signed.

4.3 Exclusion criteria

1) diagnosis of cryptococcosis or other systemic fungal infections; 2) a history of a known allergy or intolerance to miconazole nitrate and/or itroconazole; 3) use of rifampicin, rifabutin, isoniazid, phenobarbital, phenytoin, methylprednisolone, carbamazepine, terfenadine, astemizole or cisapride; 4) history of pregnancy or breast-feeding; 5) history of psychological disorder that enable to cooperate; 6) hepaticinsufficiency with serum aminotransferase and total bilirubin levels at 1.5 times the upper limit of normal, or active clinical signs 2 months prior to the trial; 7) serum creatinine levels at 1.5 times the upper limit of normal; 8) history of cardiac insufficiency such as ischemic heart failure; 9) history of hematological diseases; 10) use of systemic or topical antifungal therapy within 2 weeks before study entry ( patients received topical treatment with miconazole or nystatin cream or suppositor remain eligible for enrollment in the study); 11) diagnosis of chronic mucocutaneous candidiasis; 12) hyposalivation related disease or drug-taking; 13) HIV positive; 14) history of participating other clinical trials within 4 weeks before study entry.

4.4 Recruitment

Patients will be recruited from nine dental hospitals from northern, southern, western and eastern China. The study will be staffed with a clinical research team, who will inform potential participants about the aims, methods and potential risks and benefits of this study. All the investigators from the nine centers will be trained prior to the initiation of the study. Participants will be informed that their participation is voluntary and that choosing not to participate or withdraw will not affect their care. Patients unable to understand or follow the study procedures will not be enrolled. Potential participants will be recruited to the study following signing the written informed consent including information about the trial.

**5. Index of efficacy**

All subjects involved were assigned to treatment group receiving 10mg miconazole mucoadhesive tablet for topical use once a day or control group receiving itroconazole capsule 100mg QD for a treatment period of 2 weeks and followed up for 2 week. Primary outcome measures include the evaluation of clinical symptoms (pain and burning sensation rating), signs (pseudomenbran and erythema grade) candida elimination rate. Secondary outcome measures include ameliorative rate of symptoms, occurrence of adverse events and total usage of other therapies as recorded in the participants’ daily diary and laboratory studies which include blood pressure, electrocardiography, full blood count and liver and kidney function tests.

5.1 Primary outcome measurements

Clinical cure (short and long term): clinical symptoms (pain and burning sensation rating), signs (pseudomenbran and erythema grade)

5.2 Secondary outcome measurements

• Clinical improvement rate: (score of symptom and clinical sign at baseline- score of symptom and clinical sign post treatment)/ score of symptom and clinical sign at baseline

• Mycological: Eradication of organism (negative culture and negative microscopy)*

• Safety: Number of patient withdrawals due to adverse reactions; Incidence of adverse reactions (reported as side-effects).

*Candida albicans is found routinely in asymptomatic population, therefore, it would have been inappropriate to use mycological eradication as the primary outcome measure for the study.

The cure is defined as complete clinical response and eradication of Candida spp. at Day 7 or Day 14. A complete clinical response was defined as the complete disappearance of oral candidiasis lesions, and a partial clinical response was defined as ≥60% of clinical improvement rate compared with the score at baseline. <60% of clinical improvement rate and candida persistence, re-infection or species shifts were considered failures. Finally, biologic, local, and general safety was compared between treatments.

Participants will also be required to complete their daily diaries from the start of the treatment period until the end of the treatment period. Blood tests for full blood count and liver and kidney function tests will be conducted at initial assessment and during the final clinic visit (week 4).

5.3 Safety monitoring

A blood sample will be obtained at baseline（day -3~0）, endpoint of 2-week treatment (day 14+1), 2-week follow-up visit （If the patient reported an abnormal blood result at 3rd visit, then additional blood test would be required in the 4th visit）. A full blood examination, assess liver and renal function. For the duration of the trial, the principal investigator will be contactable by telephone at all times. Any adverse medical events reported by the patient will be recorded in the patients’ medical records at each visit. All adverse events will be followed up from the date it is brought to the investigator’s attention until the adverse event has been resolved. In the occasion of a severe adverse event occurring after initiation of the trial, the event will be recorded and immediately reported to the SFDA, followed by a detailed written report. Participants will be identified by their ID codes to maintain confidentiality. Participants may be withdrawn from the trial by the investigator if a serious adverse event occurs.

**6. Trial discontinuation or modification**

The participants will be able to end participation or withdraw consent at their own request at any time. Participants will be withdrawn from the trial if they suffer from an unexpected serious adverse event.

Under some circumstances, certain modifications to the manufacturing changes, and/or to the clinical protocol during the course of the clinical investigation can be made. Any such changes will be required to report to IRB for approval.

6.1 Withdraw decided by researcher

Subject withdrew the study indicates that the enrolled subject cannot continue research at certain condition, and researcher decides the subject to withdraw from research process.

6.2 Subject withdraw research voluntarily

According to informed consent, subject has the right to withdraw research; if the subject does not put forward the idea of withdrawing research clearly, but losses to follow up for refusal taking medication or detection, he or she also withdraws researcher (or “off”). The reason of withdraw should be comprehended and recorded.

The history record table should be preserved for the subject who withdraws research for any reasons, the last detection result can be considered as final result, and curative efficacy and adverse effect should take full data set analysis.

6.3 Situation to discontinue research

Research suspension refers to the clinical research discontinues during process, not ends as planned. The purpose of research suspension is to protect interests of subjects, ensure quality of research and avoid unnecessary economic losses.

(1) If there is serious security problem, the research must discontinue immediately.

(2) If the drug is with poor curative efficacy, or even ineffective, or without clinical significance, the research should discontinue, to avoid delay for effective treatment of subjects or unnecessary economic loss.

(3) If the clinical research program has a major mistake, and is difficult to evaluate drug effects; or a better designed program has significant deviation during implementation, and cannot continue to carry out to evaluate drug efficacy, the research should discontinue.

6.4 Situation to early stop research

The enrollment may end early when target sample size were achieved.

**7. Investigating drugs**

7.1 Name, source of investigational drug

1. Miconazole 10mg mucoadhesive tablet (Tibotec Pharmaceuticals Ltd.，Shenzhen, China)

2. Itraconazole 100mg capsule (Xian Janssen Pharmaceutical Ltd., Xi’an, China)

7.2 Drug inventory and recovery

At each visit, observe doctor should record receive amount, dosage and return amount of drug in detail, to determine the compliance of patient, which should be recorded in case report table.

7.3 Drug preservation

Investigational drug should be locked and kept in safe and controllable indoor areas, and keep dry. Each center should specify a medical management person to keep and manage drugs.

7.4 Drug dispensation and recovery

After passing screening, subjects should be given drugs by researcher in order. At each dispensation, researcher should immediately and accurately fill in drug dispensation record, record amount of dispensed drugs, and remind subjects taking remain drugs back at the next visit; the researcher should recover and check the amount of recovery drugs, which should be recorded in drug dispensation/ recovery record. After research, all remained drugs should be returned to sponsor, and recorded in recovery table of investigational drug.

7.5 Drug inventory and management

At each follow-up, observe doctor should record faithfully and in detail whether the patient takes treatment on time at designated place, to determine the degree of subject compliance. The record should be made at format medical record. Medication compliance=total amount of actual drug use/ total amount of required drug use by program ×100%. At completion, research drug administrator is responsible for giving back remaining drugs to biding unit or destroying remaining drugs. Research drugs should be preserved in locked specific refrigerator in 4 ºC.

**8. Therapeutic Regimen and Research Process**

8.1 Dosage regimen

Trial group: Miconazole 10mg mucoadhesive tablet was administered topically once daily, with the rounded side of the tablet applied to the canine fossa in the morning after brushing the teeth.

| Step 1 | 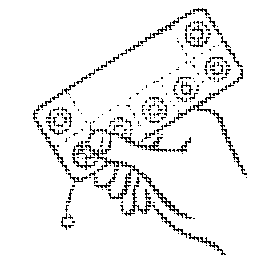 | Take the patch out from the package. |
| --- | --- | --- |
| Step 2 | 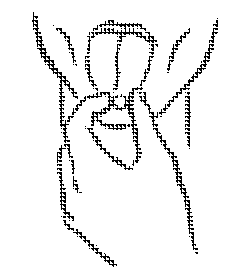 | Moisten the patch with tongue. |
| Step 3 | 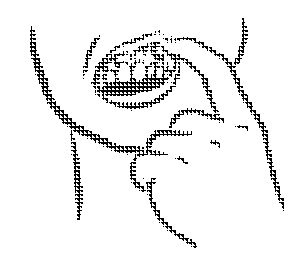 | Put the patch on the upper gingiva adjacent to canine with finger. |
| Step 4 | 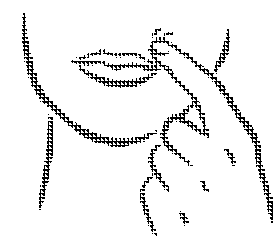 | Patch is held in place for 30 s with slight pressure. Do not lick with tongue for the first a few minutes to avoid removal. |

Figure 1. Application of miconazole mucoadhesive tablet.

Control group: Itraconazole capsule 100mg once daily, taken immediately after a full meal due to improvement of absorption.

8.2 Administration Instructions

Miconazole mucoadhesive tablet should be applied in the morning, after brushing the teeth. The tablet should be applied with dry hands. The rounded side surface of the tablet should be placed against the upper gum just above the incisor tooth (canine fossa) and held in place with slight pressure over the upper lip for 30 seconds to ensure adhesion. Either side of the tablet can be applied to the gum.

Once applied, miconazole mucoadhesive tablet stays in position and gradually dissolves. Before applying the next tablet, the patient should clear away any remaining tablet material. Food and drink can be taken normally when miconazole mucoadhesive tablet is in place but chewing gum should be avoided.

If miconazole mucoadhesive tablet does not adhere or falls off within the first 6 hours, the same tablet should be repositioned immediately. If the tablet still does not adhere, a new tablet should be placed. If miconazole mucoadhesive tablet is swallowed within the first 6 hours, the patient should drink a glass of water and a new tablet should be applied only once.If miconazole mucoadhesive tablet falls off or is swallowed after it was in place for 6 hours or more, a new tablet should not be applied until the next regularly scheduled dose.

8.3 Research process

Potential participants will undergo preliminary screening for eligibility during visit 1 by investigators, which will include a registered oral medicine specialist. Eligible participants will then undergo initial assessments for review of system (ROS), baseline data collection, which include the symptom scoring, grade of clinical sign of oral candidiasis, measurement of vital signs (temperature, blood pressure and heart rate. ECG), full blood count, kidney function test and liver function test. The candida-related laboratory testing (smear test and fungi culture) will be performed to gather the baseline data of candida infection and colony forming unit count. A daily diary will then be given to record the change of symptoms and occurrence of adverse events and use of topical treatments during the 2-week treatment period. After signing the consent form, participants will be randomly assigned to either the treatment (miconazole mucoadhesive tablet) group or the control (itroconazole capsule) group and the treatment period will start. During this clinic visits, participants will be given 1 weeks’ worth of miconazole mucoadhesive tablet or itroconazole capsules and daily diaries for the fortnight.

In visit 2 of 1week after, the participants will be re-evaluated on the symptom scoring, grade of clinical sign, vital signs, smear test and fungi culture). The participants will repeat 1-week treatment and record the daily diary during the following week. In visit 3 (the following day after the 2-week treatment period) and visit 4 (2-week follow up after treatment ceasing), the participant will be evaluated by performing clinical and laboratory examination as baseline.

Throughout the treatment period, other medication or therapies, such as anti-hypertension drugs, is allowed to be used on an ‘as needed’ basis. During this treatment period, participants will also be required to record their medication intake, including trial interventions, other medications intake and occurrence of adverse events to assist with compliance monitoring and acceptability of intervention. Participants will be asked to return their medication pack to enable the counting of left-overs as well as part of participant adherence monitoring.


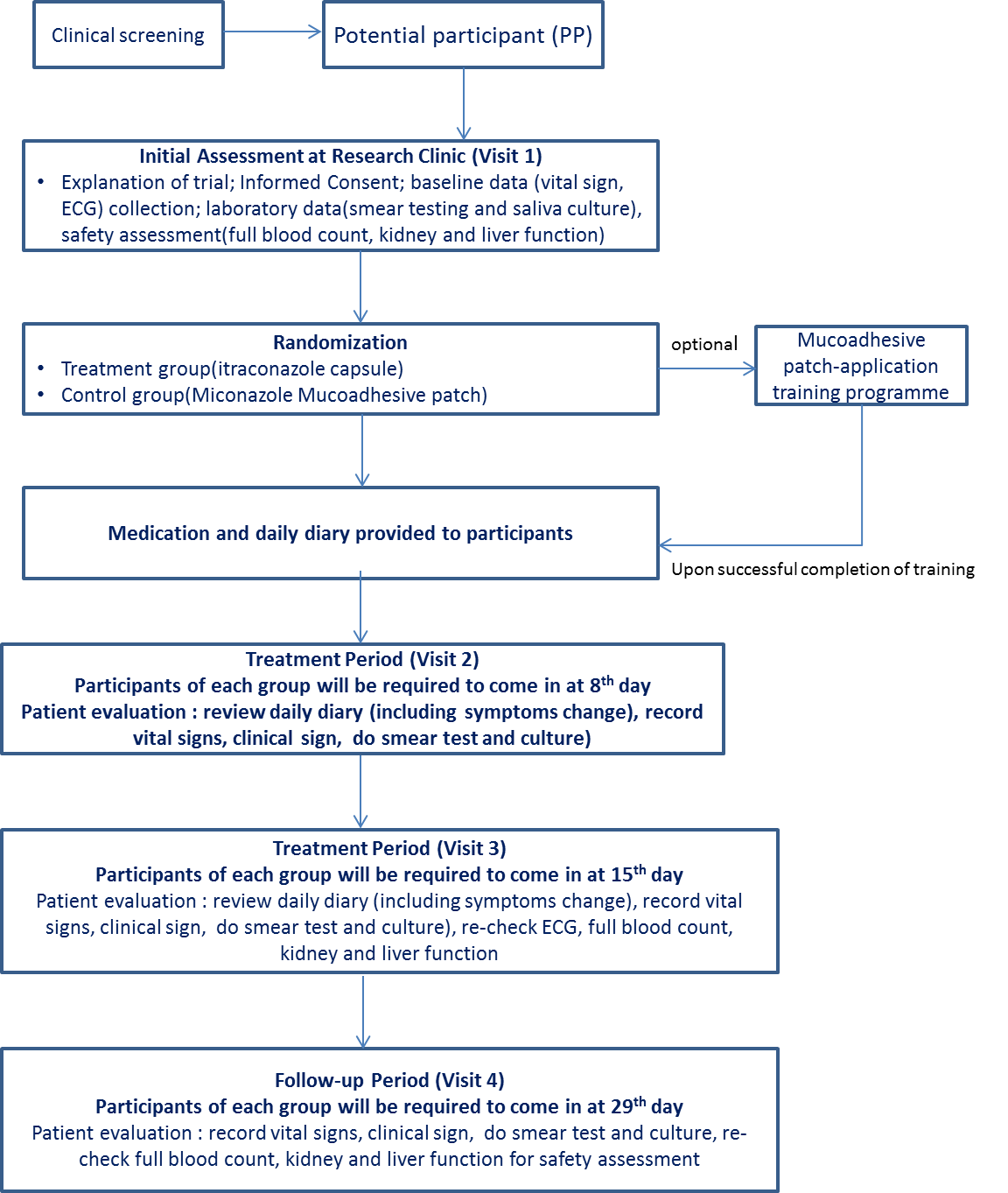


Figure 2. Outline of trial protocol

**9. Observation of Adverse Event**

9.1 Definition

(1) Adverse event (AE): any unfavorable medical incident happens since the time when the subject signs the informed consent and enrolls the trial to the finish of the last follow-up is judged as adverse event, no matter whether the medical incident is associated with the investigational drug or not.

(2) Significant adverse event: besides severe adverse event, any adverse event happens and causes the use of pointed medical measures (e.g. drug withdrawal, reducing dosage and expectant treatment) and hematological and/or other laboratorial anomaly.

9.2 Criteria for Judging Intensity of Adverse Event

All clinical adverse events occurred in this clinical research will be recorded on the adverse events page of CRF and classified according to the intensity. In order to have a unified standard, the intensity grade of events is as follows:

Slight perceptible malaise but not affect daily activities

Moderate stronger malaise leading to affect or reduce daily activities

Severe malaise that unable to work or carry out daily activities

It needs to pay attention to distinguish the severity and intensity of adverse events. Severity is used to describe intensity and it may not be a serious adverse event (SAE).

9.3 Judging standard of the relation between adverse events and investigational drug

The causality analysis of the relation between all adverse events and the investigational drug is judged according to five grades, i.e. certainly related, probably related, possibly related, possibly unrelated and definitely unrelated. The first three grades are judged drug adverse reaction. In the causality analysis we consider the following five aspects.

1) Do the time of beginning to use the medicine and the time of appearance of Adverse Drug Reaction (ADR) have reasonable precedence relation (appearance during using medicine)?

2) Does the suspected ADR match the already-known ADR of the drug (conformity with the literature)?

3) Can the suspected ADR be explained by influences of combining with drug, original drug, clinical state of patients or the effect of other therapies (other explanations)?

4) Do the suspicious ADR disappear or reduce after drug withdrawal or decrement (withdrawal symptom)?

5) Does suspicious ADR appear again after taking the same medicine (taking again, appearing again)?

9.4 Abnormal laboratory results

The investigator should judge whether the abnormal laboratory results are of clinical significance and provide possible interpretation. The reported adverse event which causes the abnormal laboratory results should also be recorded as adverse events in ADR.

- 9.5 Follow-up and adverse event record

Adverse events, especially the events which have a connection with the investigational drugs, should be followed up until they return to the baseline or tend to be steady. If follow-up can’t make the adverse events back to the baseline or to be steady, then it should be recorded and explained in the CRF. Any serious adverse events occurred in the clinical trial must be reported to the sponsor and major research institution within 24 hours. At the same time, the investigator must fill in the SAR report and record the  occurrence time, seriousness, duration, measurement and turnover.

**10. Data management**

Researchers should load original observation record of subject into case report table  correctly, completely, clearly and timely. Supervisor should confirm that the trail is carried out according to trail scheme, and all case report tables are filled correctly and completely, the data is consistent with original materials. If there is any mistake or omission, researcher should correct it immediately. After being checked, the case report table should be submitted to data administrator of clinical trial by supervisor.

If there is any problem, data administrator should inform supervisor immediately,  and ask researcher to make an explanation. Query table should be used to record various questions and answers, and kept properly for reference.

**11. Statistical Analysis**

11.1 Analysis set

Full analysis set (FAS): it refers to the data set of all subjects participating in

randomization and being evaluated after medication at least once. For those subjects who fail to observe all healing effect evaluation, we conduct the data transfer according to LOCF (last observation carry forward) principle.

Per protocol set (PPS): it refers to the data set of all subjects meeting inclusion criteria, inconsistent with exclusion criteria and completing therapeutic scheme. Analysis (per protocol, PP) is taken on data of all cases consistent with research scheme, with good dependency and completion of filling in contents specified by CRF.

Safety set (SS): It refers to the data set of subjects who are grouped at random, take the research drug and at least have one safety assessment after the baseline.

Baseline comparative analysis and curative effect analysis are conducted based on

FAS and PPS, and safety evaluation is conducted according to SS.

11.2 Statistical analysis method

Analysis and reporting of the results will follow the CONSORT guidelines for reporting randomised controlled trials. All participants who had at least 1 efficacy evaluation after randomization will be included in the analysis. Safety analyses included all patients who received at least 1 dose of study medication. The trial data will be processed and analyzed by an independent statistician, who will be blinded to participant allocation.

A non-inferiority statistical approach was used. Continuous data were compared using either the Student t test or the Mann-Whitney nonparametric test. For binary data, the 2 treatment groups were compared using the chi-square test or the Fisher exact test. The Mantel-Haenszel test was used to evaluate ordinal quantitative data.

**12. Ethics**

12.1 Ethics review

This clinical study must follow Helsinki Declaration and related clinical research laws and regulations in China. Before research, Ethics Committee of research responsible hospital should review research scheme, and implement research scheme after signing approval documents. Suggestions of Ethics Committee should be: agree, which refers to consent after taking necessary correction; disagree, which refers to terminate or suspend approved research.

12.2 Informed consent for subjects

Before being enrolled in the research, each patient or the designated representatives should be introduced about objectives, characteristics, procedures and possible benefits and risks of the research by doctor completely and comprehensively. The patient has right to withdraw research at any time. Each patient is given a written informed consent (as an appendix in scheme) before research, and agrees its content after understanding. After signing Informed Consent, the patient can be enrolled in clinical study. As original clinical research data, the Informed Consent should be preserved for reference.

12.3 Ethics and trial registration

Written informed consent is required prior to participation from each participant or their guardian, and they will be informed of the nature of this trial including its purpose, procedures involved, expected duration, potential risks and discomfort, as well as the possible benefits they will receive from it. There is no time limit for them to ask related questions and respond to the invitation to participate. Participants will be also informed that they are free to withdraw from the study at any stage for any reason and their personal information will be undisclosed and kept securely.

The trial has also been registered with the Chinese Clinical Trial Registry (ChiCTR-TRC-13003935).

**13. References**

1. Pappas, P.G., et al., Clinical practice guidelines for the management of candidiasis: 2009 update by the Infectious Diseases Society of America. Clin Infect Dis, 2009. 48(5): p. 503-35

2. Moher D, Hopewell S, Schulz KF, Montori V, Gøtzsche PC, Devereaux PJ, Elbourne D, Egger M, Altman DG: CONSORT. CONSORT 2010 explanation and elaboration: updated guidelines for reporting parallel group randomised trials. Int J Surg 2012;10: 28–55.
